# Using a rapid environmental scan methodology to map country-level global health research expertise in Canada

**DOI:** 10.1186/s12961-020-0543-x

**Published:** 2020-04-09

**Authors:** Ranjana Nagi, Susan Rogers Van Katwyk, Steven J. Hoffman

**Affiliations:** 1 Global Strategy Lab, Dahdaleh Institute for Global Health Research, Faculty of Health, and Osgoode Hall Law School, York University, 4700 Keele Street 2120 Dahdaleh Building, Toronto, Ontario M3J 1P3 Canada; 2 School of Epidemiology and Public Health, Faculty of Medicine, University of Ottawa, Ottawa, Ontario Canada; 3 Department of Global Health and Population, Harvard T.H. Chan School of Public Health, Harvard University, Boston, MA United States of America; 4 Department of Health Research Methods, Evidence and Impact and McMaster Health Forum, Faculty of Health Sciences, McMaster University, Hamilton, Ontario Canada

**Keywords:** Global health, Meta-research, Capacity-building, Canada

## Abstract

**Background:**

Many countries are currently rethinking their global health research funding priorities. When resources are limited, it is important to understand and use information about existing research strengths to inform research strategies and investments and to drive impact. This study describes a method to rapidly assess a country’s global health research expertise and applies this method in the Canadian context.

**Methods:**

We developed a three-pronged rapid environmental scan to evaluate Canadian global health research expertise that focused on research funding inputs, research activities and research outputs. We assessed research funding inputs from Canada’s national health research funding agency and identified the 30 Canadian universities that received the most global health research funding. We systematically searched university websites and secondary databases to identify research activities, including research centres, research chairs and research training programmes. To evaluate research outputs, we searched PubMed to identify global health research publications by Canadian university-affiliated researchers. We used these three perspectives to develop a more nuanced understanding of Canadian strengths in global health research from different perspectives.

**Results:**

Canada’s main global health research funder, the Canadian Institutes of Health Research, invested a total of $314 M from 2000 to 2016 on global health research grants. This investment has contributed to Canada’s wealth of global health research expertise, including 12 training programmes, 27 Canada Research Chairs, 6 research centres and 30 WHO Collaborating Centres across 27 universities. Research activities were concentrated in Canada’s biggest cities and most commonly focused on health equity and globalisation issues. Canadian-affiliated researchers have contributed to a research output of 822 unique publications on PubMed. There is an opportunity to build global health expertise in regions not already concentrated with research activity, focusing on transnational risks and neglected conditions research.

**Conclusions:**

Our three-pronged approach allowed us to rapidly identify clear geographic and substantive areas of strength in Canadian global health research, including urban regions and research focused on health equity and globalisation topics. This information can be used to support research policy directives, including to inform a Canadian global health research strategy, and to allow relevant academic institutions and funding organisations to make more strategic decisions regarding their future investments.

## Background

The global health research funding landscape is complex, with multiple public and private groups that vary greatly in their financing capacities and research interests. Despite the variation, a large proportion of health research funding comes from funding bodies in high-income countries [1]. As national priorities shift and funders explore new ways of setting research agendas [2], it is important that strategies are informed by knowledge of the relevant jurisdiction’s current strengths and future opportunities. Building on strengths is particularly beneficial when aiming to maximise limited research funding [3] and identifying existing research strengths in global health can assist countries in effectively leveraging these domestic assets in their foreign policy and international development efforts [4].

**Fig. 1:**
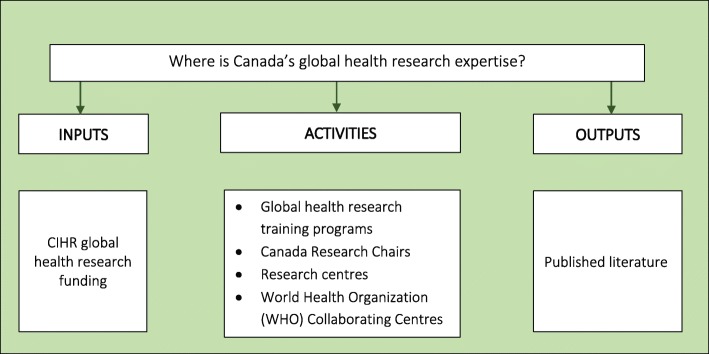
Inputs, activities and outputs in global health research in the Canadian context

**Fig. 2:**
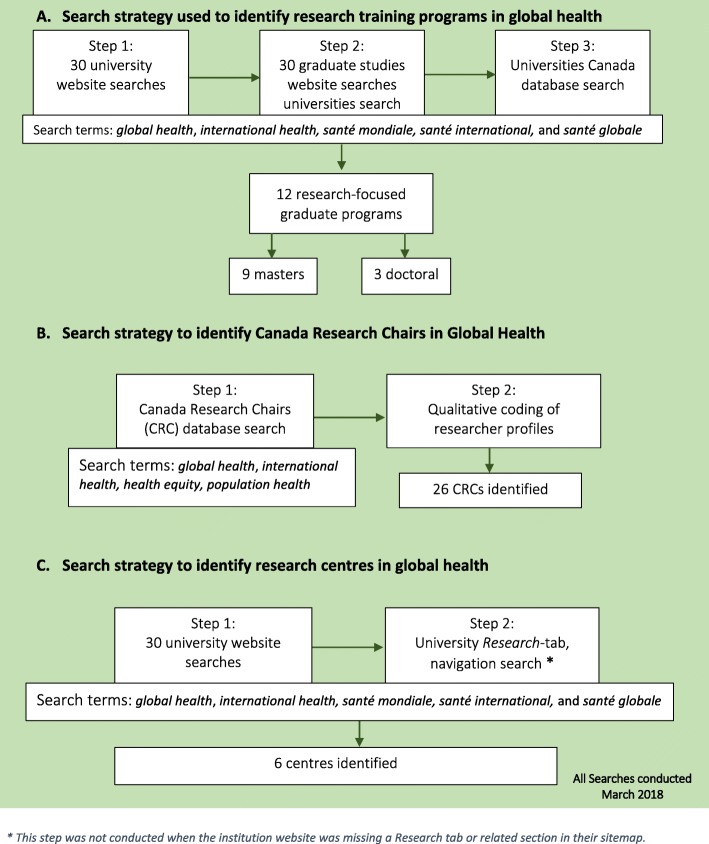
Search strategies used to identify research training programmes, Canada Research Chairs in global health and research centres. *This step was not conducted when the institution website was missing a Research tab or related section in their sitemap

In the Canadian context, research funders are estimated to collectively invest C$90 million per year in global health research activities [5]. Yet, despite this sum, the Canadian funding landscape for global health has been criticised for being uncoordinated and inefficient [5]. Box 1 describes the roles of Canada’s main global health funders; however, as a ‘middle power’, Canada cannot meaningfully invest in all areas of global health research concomitantly. Identifying existing strengths in research expertise may prove difficult, especially for countries with limited resource capacity. Despite the range of literature on programme implementation and evaluation frameworks, to the best of our knowledge, there is no existing framework that can be used to capture research expertise at the national level. We sought to fill this gap by developing a framework that provides a systematic approach to identifying expertise by drawing upon a range of research inputs, activities and outputs. In this manuscript we aimed to (1) summarise and critically evaluate the available evidence on global health research expertise at Canadian universities through research inputs, activities and outputs (Fig. 1), and (2) assess Canada’s overall global health research expertise using strengths in select research inputs, activities and outputs [6, 7].

Box 1. Global health funding bodies in Canada **Global health in Canada is predominantly funded by three agencies:**

**1. Canadian Institutes of Health Research:** a federal funding agency that supports health research across four pillars (biomedical, clinical, health systems services and population health) [8]

**2. International Development Research Centre:** a Canadian Crown corporation that supports research in developing countries as a part of Canada’s foreign affairs and development efforts [8]

**3. Grand Challenges Canada:** a non-profit receiving financial support primarily from the government through Global Affairs Canada, funds innovators in low- and middle-income countries and Canada [9, 10]

## Methods

We developed a three-pronged analytic framework that includes research inputs, activities and outputs. For this assessment, research ‘inputs’ were global health research funding from Canadian Institutes of Health Research (CIHR) – the Canadian funder with the largest focus on domestic global health research capacity. Research ‘activities’ included research training programmes in academic institutions, programmes aimed at recruiting top research talent in the country (e.g. Canada Research Chairs (CRC) Program), research centres at these institutions and formal research partnerships with multilateral health organisations (e.g. WHO Collaborating Centres). To varying degrees, these activities contribute to research ‘outputs’ such as published literature [11–13].

### Study definitions, sample and qualitative coding

We defined global health research as research focusing on health, health systems, health inequities and health policy challenges facing populations living in conditions of vulnerability in both low- and middle-income countries (LMICs) and high-income countries [7]. We identified research activities by systematically searching for research training programmes, research centres and research chairs focused on global health. We mapped research outputs by identifying highly cited authors, their affiliated universities and their areas of research from PubMed. For research inputs, we systematically analysed an administrative dataset of global health research funding in Canada from 2000 to 2016, specifically focusing our analysis on the 30 research institutions in Canada that received the most global health research funding from CIHR over this 15-year period [7].

Database and web searches were conducted in March 2018. When applicable, we coded activities and outputs using a previously developed system of 13 research areas and four research themes (Box 2), which are consistent with the CIHR’s mandates, research pillars and institutes [7, 14]. When activities and outputs pertained to multiple foci, they were coded using the most-fitting primary focus. Activities and outputs were coded as ‘other’ when there was inadequate information.

### Identifying research activities

#### Global health research training programmes

We defined research training programmes as any research-focused graduate education programmes that explicitly focused on global health. An explicit focus on global health was indicated by the words ‘global health’ or ‘international health’ in the name of the programme, or any of its concentrations or specialisations. Indicators of a research-focused programme (as opposed to a professional focus) included a requirement or option to complete a thesis or major research project (as opposed to an internship, practicum or coursework-only programme)*.*

We conducted three sets of systematic searches (Fig. 2) to identify global health research training programmes. The first systematic search focused on the main websites of the 30 most funded universities from our dataset of CIHR funding, while the second focused on the specific graduate studies websites of all 30 universities. Both types of websites were searched for the following terms: ‘global health’, ‘international health’, ‘*santé mondiale*’, ‘*santé internationale*’ and ‘*santé globale*’*.* The first 100 results of each search were manually reviewed; this number was selected to balance the relevance of the result to the research question and screening feasibility. To supplement internet searches, a third systematic search used an administrative database on Canadian university programmes – the Universities Canada Database [15]. The aforementioned terms were used to search this database as well. The combined results of these searches were revisited and available programme descriptions were consulted to yield a list of global health research training programmes.

#### Canada Research Chairs in Global Health

The Canada Research Chairs (CRC) Program is a federal initiative that funds 2000 university professorships to attract and retain top research talent [16, 17]. We searched profiles in the CRCP Database for individuals conducting global health research using the following terms: ‘global health’, ‘international health’, ‘population health’ and ‘health equity’. This database contained information such as the Chair’s name, title, tier, host university, biography and research discipline.

#### Research centres and WHO Collaborating Centres

Global health research centres were defined as organised groups or units conducting research on global health that have been officially recognised as such by their host universities. We conducted two sets of systematic searches of the 30 universities’ websites to identify relevant centres (Fig. 2). First, we searched the internal websites of these universities using the same key terms as our searches for research training programmes and reviewed the first 100 results. Second, we conducted an additional supplementary search whereby we sought out the ‘Research’ tab in the site navigation area of each university’s website. This tab led to webpages that listed all research centres hosted by the university. We reviewed the lists available on these web pages to identify centres that conducted global health research. WHO Collaborating Centres are institutions or groups that conduct activities in support of WHO’s various initiatives [18]. Designated by WHO’s Director-General, Collaborating Centres can often partake in activities like research, training and education, advising WHO staff on specific topics, and collecting and collating information and resources [18]. We searched the WHO Collaborating Centres Database for information on the title of the centre, the designated parent institution (i.e. usually a university), the subjects of focus and the types of activities conducted [18, 19]. This information allowed us to understand which Canadian universities were involved and in what capacity, including which subject areas and what types of activities.

### Identifying research outputs

#### Prominent focus areas of publications

We searched PubMed using a structured search query combining the Medical Subject Heading (MeSH) term ‘Global Health’ and Author Affiliation ‘Canada’ for articles published recently, from January 1, 2013, to March 1, 2018. We exported the search results and qualitatively coded abstracts to identify the foci of each global health research publication involving Canadian-affiliated authors as either as primary authors or as co-authors [7].

#### Quantity of publications at 30 universities

We conducted an additional search of PubMed for publications from April 1, 2000, to March 31, 2016, to identify the quantity of global health publications produced by authors affiliated with the 30 universities. We used this time period to match the fiscal-year funding period data provided by CIHR. In these searches, we used the Global Health MeSH term and, for the Affiliation field, we searched the names of each of the 30 universities.

## Results

Our search shows that universities have developed their capacity in global health research to varying degrees. Canadian universities have a diverse range of global health research activity – 12 global health research training programmes, 26 CRCs in global health, 6 research centres in global health and 30 WHO collaborating centres (Table 1). Canadian global health research outputs include 822 unique citations with a Global Health MeSH in the PubMed database, with authors primarily belonging to universities in urban areas and research topics primarily focusing on ‘health equity’ and ‘globalization’.

**Table 1:** The 30 Canadian universities receiving the most Canadian Institutes of Health Research global health research funding

Institution name	Location	Input	Activities				Outputs
		Funding amount, 2000–2016 (*n* = $314,130,951)	Research training programmes (*n* = 12)	Canada Research Chairs in global health (*n* = 26)	Research centres (*n* = 6)	WHO Collaborating Centres (*n* = 30)	Publications with Global Health MeSH (*n* = 1069)
Acadia University	Wolfville, NS	$196,000	0	0	0	0	0
Brock University	St. Catherine’s, ON	$157,285	0	0	0	0	1
Carleton University	Ottawa, ON	$114,954	0	0	0	0	5
Dalhousie University	Halifax, NS	$5,725,223	0	4	0	1	32
McGill University	Montreal, QC	$47,881,835	1	2	0	1	127
McMaster University	Hamilton, ON	$30,759,199	1	1	0	2	128
Queen’s University	Kingston, ON	$3,266,766	0	0	0	0	28
Ryerson University	Toronto, ON	$609,017	0	0	1	0	4
Saint Mary’s University	Halifax, NS	$105,000	0	3	0	0	1
Simon Fraser University	Burnaby, BC	$5,803,161	1	1	0	0	31
Université de Montréal	Montreal, QC	$29,678,941	2	0	0	0	28
Université de Sherbrooke	Sherbrooke, QC	$418,084	0	0	0	1	2
Université du Québec	Quebec City, QC	$730,590	0	0	0	0	4
Université du Québec à Montréal	Montreal, QC	$667,703	0	0	0	1	2
Université Laval	Quebec City, QC	$27,934,610	1	0	0	0	16
University of Alberta	Edmonton, AB	$7,931,583	2	0	0	1	71
University of British Columbia	Vancouver, BC	$37,423,959	0	4	1	1	111
University of Calgary	Calgary, AB	$4,175,934	0	0	0	1	60
University of Guelph	Guelph, ON	$534,900	0	0	0	0	7
University of Lethbridge	Lethbridge, AB	$100,000	0	2	1	0	4
University of Manitoba	Winnipeg, MB	$24,044,245	0	4	0	0	31
University of Ottawa	Ottawa, ON	$14,969,455	0	2	1	1	99
University of Prince Edward Island	Charlottetown, PEI	$168,750	0	1	0	0	0
University of Regina	Regina, SK	$1,208,518	0	0	0	0	3
University of Saskatchewan	Saskatoon, SK	$3,646,490	0	0	0	0	17
University of Toronto	Toronto, ON	$48,370,150	2	4	1	4	279
University of Victoria	Victoria, BC	$2,354,077	0	0	0	0	14
University of Waterloo	Kitchener, ON	$12,090,327	0	0	0	0	12
University of Western Ontario	London, ON	$2,308,191	2	0	1	0	17
York University	Toronto, ON	$756,000	0	0	1	0	62

Funding data obtained from Canadian Institutes of Health Research. Publication counts include duplicates whenever records were co-authored by researchers at more than one of the 30 universities; there were 822 unique publications

### Global health research training programmes

We identified 12 global health research training programmes at 8 universities as described in Table 2; 4 universities – located in Toronto, Montreal, Alberta and London – hosted two programmes each, while the other 4 universities each hosted a single programme. Among the 12 programmes, 9 were Masters degree programmes, while 3 were doctoral programmes.

**Table 2:** Global health research training programmes and research centres in Canada

**Research training programmes**			
**Programme title**	Programme level	University	Location
MSc in Public Health (Global Health Option)	Masters	McGill University	Montreal, QC
MSc in Global Health	Masters	McMaster University	Hamilton, ON
MSc in Global Health	Masters	Simon Fraser University	Burnaby, BC
MSc en santé publique (option de santé mondiale)	Masters	Université de Montréal	Montreal, QC
MPH en santé mondiale	Masters	Université Laval	Quebec City, QC
MPH in Global Health	Masters	University of Alberta	Edmonton, AB
MSc in Global Health	Masters	University of Alberta	Edmonton, AB
Masters Collaborative Specialization in Global Health	Masters	University of Toronto	Toronto, ON
Masters Collaborative Program in Global Health Systems in Africa	Masters	University of Western Ontario	London, ON
PhD en santé publique (de l’option “santé mondiale“)	Doctoral	Université de Montréal	Montreal, QC
Doctoral Collaborative Specialization in Global Health	Doctoral	University of Toronto	Toronto, ON
Doctoral Collaborative Program in Global Health Systems in Africa	Doctoral	University of Western Ontario	London, ON
**Research centres**			
**Title**	Director(s)	University	Location
Centre for Global Health and Health Equity	Vahabi, Mandana & Guruge, Sepali	Ryerson University	Toronto, ON
Global Health Research Program	Spiegel, Jerry & Yassi, Annalee	University of British Columbia	Vancouver, BC
Centre for Global Public Health	Blanchard, James	University of Manitoba	Winnipeg, MB
Centre for Global Health	Tugwell, Peter	University of Ottawa	Ottawa, ON
Institute for Global Health Equity and Innovation	Jadad, Alex	University of Toronto	Toronto, ON
Dahdaleh Institute for Global Health Research	Orbinski, James	York University	Toronto, ON

Data retrieved from institution website searches and database searches in March 2018

### Canada Research Chairs in Global Health

Searching the CRC Database identified 26 individual chairs in different research disciplines at 10 universities and in 1 non-sample university (Table 3). Most CRCs in this search are at institutions in Montreal, Toronto, Vancouver and Ottawa, with 5 chairs in Montreal and 4 each in Toronto, Vancouver and Ottawa.

**Table 3:** Canada Research Chairs in global health

Chairholder	Chair title	Tier	University	Recruited from	Research foci
1. Brockman, Mark	Viral Pathogenesis and Immunity	2	Simon Fraser University	Harvard Medical School, USA	Neglected Conditions
2. Chan, Laurie	Toxicology and Environmental Health	1	University of Ottawa	Domestic	Globalisation
3. Forman, Lisa	Human Rights and Global Health Equity	2	University of Toronto	Domestic	Health equity
4. Frank, Erica	Preventive Medicine and Population Health	1	University of British Columbia	Emory University School of Medicine	Other
5. Grimshaw, Jeremy	Health Knowledge Transfer and Uptake	1	University of Ottawa	University of Aberdeen, UK	Other
6. Jha, Prabhat	Global Health	1	University of Toronto	Domestic	Other
7. Kaufman, Jay	Health Disparities	1	McGill University	University of North Carolina at Chapel Hill, USA	Globalisation
8. Labonté, Ronald	Globalization and Health Equity	1	University of Ottawa	Domestic	Globalisation
9. Lee, Kelley	Global Health Governance	1	Simon Fraser University	London School of Hygiene & Tropical Medicine, UK	Globalisation
10. Love, Oliver	Integrative Ecology	2	University of Windsor	Domestic	Globalisation
11. Masuda, Jeffrey	Environmental Health Equity	2	Queen’s University	Domestic	Globalisation
12. Menec, Verena	Healthy Aging	2	University of Manitoba	Domestic	Health equity
13. Nandi, Arijit	Political Economy of Global Health	2	McGill University	Harvard University, USA	Globalisation
14. Nicolau, Belinda	Life Course Oral Epidemiology	2	McGill University	Domestic	Health equity
15. Price, Eric	Radiochemistry	2	University of Saskatchewan	Memorial Sloan Kettering Cancer Center, USA	Neglected conditions
16. Revie, Crawford	Population Health: Epi-Informatics	2	University of Prince Edward Island	University of Strathclyde, UK	Transnational risks
17. Rosella, Laura	Population Health Analytics	2	University of Toronto	Domestic	Neglected conditions
18. Sagan, Selena	RNA Biology and Viral Infections	2	McGill University	Stanford University, USA	Neglected conditions
19. Schechter, Martin T.	HIV/AIDS and Urban Population Health	1	University of British Columbia	Domestic	Neglected conditions
20. Sin, Don D.	Chronic Obstructive Pulmonary Disease	1	University of British Columbia	Domestic	Other
21. Tugwell, Peter	Health Equity	1	University of Ottawa	Domestic	Globalisation
22. Urquia, Marcelo	Applied Population Health	2	University of Manitoba	Domestic	Globalisation
23. Vallée-Bélisle, Alexis	Bioengineering and Bionanotechnology	2	Université de Montréal	Domestic	Other
24. Waddell, Charlotte	Children’s Health Policy	2	Simon Fraser University	Domestic	Globalisation
25. Winer, Daniel	Immunometabolism	2	University of Toronto	Domestic	Other
26. Yassi, Annalee	Global Health and Capacity Building	1	University of British Columbia	Domestic	Other

Data retrieved from the CRC Database in March 2018

The majority of these CRCs conducted research that fits under CIHR’s population health research pillar (65%) and 38% specifically focused on globalisation. The programme supports both “exceptional emerging researchers” and “*outstanding established researchers*”, given the Tier 2 and Tier 1 title, respectively [16]. More than half are Tier 2 CRCs (58%), while the remaining 42% are Tier 1 CRCs. Women made up 34% (*n* = 9) of the CRCs in global health.

### Research centres and WHO Collaborating Centres

There are 6 officially recognised groups dedicated to conducting global health research (Table 2) and 30 actively designated WHO Collaborating Centres (Table 4). As of March 2018, the city of Toronto hosted the greatest number of both research centres and WHO Collaborating Centres. Canadian WHO Collaborating Centres are mostly engaged in training and education, product development (e.g. guidelines, manuals, methodologies) and research (Table 5). The substantive issues being addressed in the Collaborating Centres vary; however, the most popular issues include environmental health and hazards (6 of 30 centres) and occupational health (6 of 30 centres).

**Table 4:** WHO Collaborating Centres in Canada

WHO Collaborating Centre in…	Director	Host institution	Location
1. Addiction and Mental Health	Shield, Kevin	Centre for Addiction and Mental Health	Toronto, ON
2. Age-friendly Cities and Communities	Garon, Suzanne	Centre hospitalier universitaire de Sherbrooke	Sherbrooke, QC
3. Bioethics	Gibson, Jennifer	University of Toronto	Toronto, ON
4. Biosafety and Biosecurity	Mantha, Stacey	Public Health Agency of Canada	Ottawa, ON
5. Classification, Terminology and Standards	Quan, Hude	University of Calgary	Calgary, AB
6. Control and Epidemiology of Rabies in Carnivores	Fehlner-Gardiner, Christine	Canadian Food Inspection Agency	Nepean, ON
7. Evidence-Informed Policy	Lavis, John N.	McMaster University	Hamilton, ON
8. Governance, Transparency and Accountability in the Pharmaceutical Sector	Kohler, Jillian	University of Toronto	Toronto, ON
9. Health Promotion	Jackson, Suzanne	University of Toronto	Toronto, ON
10. Knowledge Translation and Health Technology Assessment in Health Equity	Hatcher-Roberts, Janet	University of Ottawa	Ottawa, ON
11. Monitoring Chemical Contaminants in Food	Feeley, Mark	Health Canada	Ottawa, ON
12. Nutrition Policy for Chronic Disease Prevention	L’Abbe, Mary	University of Toronto	Toronto, ON
13. Occupational and Environmental Health	Saint-Charles, Johanne	Université du Québec à Montréal	Montréal, QC
14. Occupational Health and Safety	Jones, Gareth	Canadian Centre for Occupational Health & Safety	Hamilton, ON
15. Patient Safety and Patient Engagement	Kossey, Sandi	Canadian Patient Safety Institute	Edmonton, AB
16. Research and Training in Mental Health	Laporta, Marc	McGill University	Montréal, QC
17. Research and Training in Parasite Epidemiology and Control	Gyorkos, Theresa	McGill University	Montréal, QC
18. Safety Promotion and Injury Prevention	Maurice, Pierre	Centre de Santé publique du Québec	Beauport, QC
19. Standardization and Evaluation of Biologicals	Elmgren, Lindsay	Health Canada	Ottawa, ON
20. Studying Peri-operative Surgical Care	Cheng, Davy	University of Western Ontario	London, ON
21. Water Quality	Carreau, Greg	Health Canada	Ottawa, ON
22. Occupational and Environmental Health	Yassi, Annalee	University of British Columbia	Vancouver, BC
23. Primary Care Nursing and Health Human Resources	Baumann, Andrea	McMaster University	Hamilton, ON
24. Children’s Environmental Health	Buka, Irena	University of Alberta	Edmonton, AB
25. Environmental and Occupational Health Impact Assessment and Surveillance	Gosselin, Pierre	Centre Hospitalier Universitaire de Québec	Québec City, QC
26. Health Science Education and Practice	Morin, Martine	Université de Sherbrooke	Sherbrooke, QC
27. Health Workforce Planning and Research	Murphy, Gail T.	Dalhousie University	Halifax, NS
28. Non-Communicable Disease Policy	Rodin, Rachel	Public Health Agency of Canada	Ottawa, ON
29. Occupational and Environmental Cancer	Demers, Paul	Cancer Care Ontario	Toronto, ON
30. Occupational Health	Lazure, Louis	Institut de recherche Robert-Sauvé en santé et en sécurité du travail	Montréal, QC

Data retrieved from the WHO Collaboration Centre Database in March 2018

**Table 5:** Types of activities conducted and subjects studied by the 30 active WHO Collaborating Centres in Canada

**Type of activity conducted by WHO Collaborating Centres**	**Number of centres conducting activity (*n* = 30)**
Training and education	23
Product development (e.g. guidelines, manuals, protocols)	14
Research	10
Collection and collation of information	9
Providing technical advice to WHO	9
Information dissemination	6
Coordination of activities carried out by several institutions (e.g. other WHO collaborating centres)	4
Implementation of WHO programmes and activities at country level	3
Development and application of appropriate technology	2
Organisation of events (e.g. conferences, summits)	2
**Subject**	**Number of centres studying subject (*n* = 30)**
Environmental health and hazards other than those specifically mentioned	6
Occupational health	6
Health information, statistics, measurement and trend assessment	4
Human resources for health (excluding nursing)	4
Cardiovascular diseases	3
Health systems research and development	3
Research policy and development	3
Biological	2
Chemical safety	2
Ethics	2

Data retrieved from WHO Collaborating Centres Database in March 2018. Activity classifications and subject classifications were obtained from the WHO Collaborating Centre Database entries for each centre

### Prominent focus areas of publications

Overall, 822 unique publications were retrieved from the PubMed search covering January 1, 2013, to March 1, 2018. Of the 822 publications, 249 focused on globalisation and 244 focused on health equity. Transnational risks and neglected conditions followed with 90 and 58 publications, respectively, with an additional 183 publications coded as other. Specific research topics within these categories varied greatly. For example, the globalisation publications looked at issues like global health governance, North–South development partnerships, trade agreements and multilateral organisations [20–22]. Within health equity, publications were focused on issues pertaining to child and maternal health, with research involving sex workers, adolescents and Indigenous peoples appearing less often [23, 24]. Publications on neglected conditions included studies on malaria, polio and tuberculosis in LMIC contexts, while publications on transnational risks looked at infectious diseases with pandemic potential [25].

### Authors with Canadian affiliations and quantity of publications at 30 universities

We identified the 30 most frequently published Canadian-affiliated researchers among articles classified under the Global Health MeSH (Table 6). These 30 researchers were primary authors or co-authors on at least four or more of the 822 publications; 3 of 26 CRCs also appeared on this list. The second PubMed search of published literature yielded a total of 1069 citations. Each of these citations has at least 1 author with an affiliation at 1 of the 30 universities and the search showed that university-affiliated researchers that were situated in urban areas (Toronto and Montreal) produced the most publications classified with the Global Health MeSH term.

**Table 6:** The 30 authors with the greatest quantity of publications coded with the Global Health MeSH and Canadian affiliations from January 1, 2013, to March 1, 2018

Publications (*n* = 822)	Author’s Name	Author’s *h*-index	Author’s institutional affiliation (as listed on Scopus)	Location
11	Rehm J	14	Centre for Addiction and Mental Health	Toronto, ON
9	Yusuf S	193	Population Health Research Institute	Hamilton, ON
8	Lee K	27	Simon Fraser University, Faculty of Health Sciences	Burnaby, BC
7	Armstrong PW	100	University of Alberta, Department of Medicine	Edmonton, AB
7	Labonté R	35	University of Ottawa, School of Public Health and Preventive Medicine	Ottawa, ON
6	Cole DC	40	University of Toronto, Dalla Lana School of Public Health	Toronto, ON
6	Forman L	9	University of Toronto, Dalla Lana School of Public Health	Toronto, ON
6	Grace SL	34	York University	Vancouver, BC
6	Hoffman SJ	16	York University, Faculty of Health and Osgoode Hall Law School	Ottawa, ON
6	Lencucha R	10	McGill University, Faculty of Medicine	Montreal, QC
6	Raina P	39	McMaster University, McMaster Evidence Review and Synthesis Centre	Hamilton, ON
6	Ruckert A	10	University of Ottawa, School of Public Health and Preventive Medicine	Ottawa, ON
5	Ali U	8	McMaster University, Department of Clinical Epidemiology and Biostatistics	Hamilton, ON
5	Balion C	19	McMaster University, Faculty of Health Sciences, Department of Medicine	Hamilton, ON
5	Brown JA	13	McMaster University, Program in Evidence-Based Care	Hamilton, ON
5	Bustamam A	8	McMaster University, Department of Clinical Epidemiology and Biostatistics	Hamilton, ON
5	Clark J	4	University of Toronto, Department of Medical Imaging	Toronto, ON
5	Khan K	27	Li Ka Shing Knowledge Institute	Toronto, ON
5	McKelvie R	61	The University of Western Ontario	London, ON
5	Oremus M	18	University of Waterloo, School of Public Health and Health Systems	Waterloo, ON
5	Sohel N	14	McMaster University, Department of Clinical Epidemiology and Biostatistics^a^	Hamilton, ON
4	Atallah R	7	Jewish General Hospital, Division of Clinical Epidemiology	Montreal, QC
4	Booth RA	15	University of Ottawa, Canada, Faculty of Medicine	Ottawa, ON
4	Campbell NR	47	University of Calgary, Department of Medicine	Ottawa, ON
4	Eisenberg MJ	59	McGill University	Montreal, QC
4	Ezekowitz JA	45	University of Alberta, Canadian VIGOUR Centre	Edmonton, AB
4	Goodman SG	71	University of Toronto, St. Michael’s Hospital, Department of Medicine	Toronto, ON
4	Hill SA	24	Hamilton Health Sciences	Hamilton, ON
4	Kaplan GG	40	University of Calgary, Inflammatory Bowel Disease Clinic	Calgary, AB
4	Krahn AD	62	University of British Columbia, Heart Rhythm Services	Vancouver, BC

Information retrieved from the PubMed and Scopus databases

^a^ Sohel N did not have an affiliation listed in the Scopus database, so it was instead identified through one of their recent publications listed in PubMed

## Discussion

This study developed a three-pronged approach to map a country’s global health research strengths and expertise using research inputs, activities and outputs. The rapid environmental scan employed publicly available administrative datasets to paint a picture of the current landscape of Canada’s global health research expertise. Methods such as these can help shape policy when there is an urgent or time-sensitive need to assess strengths using an efficient, yet simple and resource-friendly approach. Our findings can continue supporting strategic and evidence-informed funding policies for Canada’s global health research stakeholders.

### Global health research in Canada

Our searches show that the main global health research strengths in Canada are health equity and globalisation. Health equity was the most common focus of research funded by CIHR grants. This research tended to focus on maternal and child health in LMICs and reflects Canada’s international development assistance priorities over the past decade [6]. Globalisation also emerged as the most common focus of CRC research interests and the focus of publications captured in the PubMed search. Future studies should explore this finding in greater detail.

**Fig. 3:**
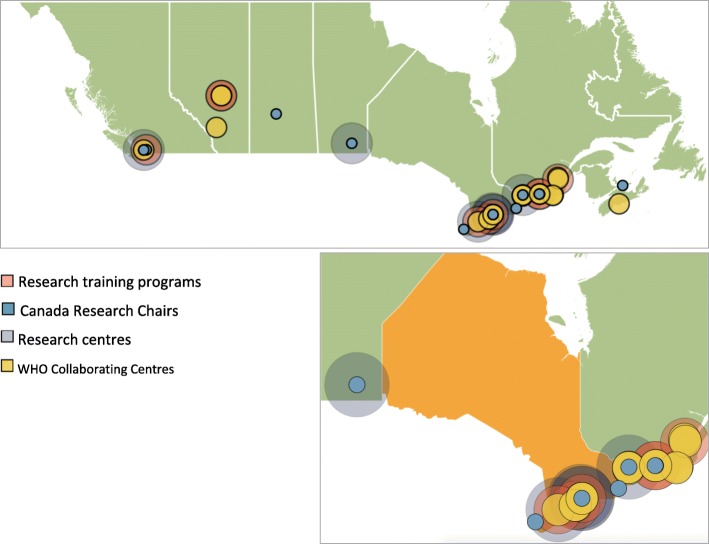
Map showing the concentration of global health research training programmes, Canada Research Chairs, research centres and WHO Collaborating Centres across Canada

Our approach suggests that Canada’s global health research expertise may be highly concentrated within a few urban areas. Based on the measures used in this scan, global health research activity appears to be flourishing in Toronto and Montreal, with the strongest contributions from 2 universities (Fig. 3). These 2 particular universities received the most CIHR global health research funding, which translated to the greatest global health research activity out of the 30 universities. We hypothesise that urban regions generally – and these universities in particular – may be benefiting from a positive feedback loop involving higher research funding levels, more CRCs, and increased research training opportunities and reputations for overall research excellence. The benefits of being situated in urban areas may include larger networks of researchers and society/government partners and better access to physical resources like high-containment biohazard labs and large teaching hospitals.

While global health research expertise is concentrated in Toronto and Montreal, at least some expertise is found in nearly all research-intensive universities across the country. Specifically, 27 of 30 universities studied here are involved in global health research through different combinations of activities. Interestingly, some universities had research centres devoted specifically to global health, but we could not identify any global health training programmes. Alternatively, universities can be less engaged with global health research training programmes, CRCs and published research contributions, while still hosting WHO Collaborating Centres.

### Research and policy implications

#### The need for a standard and operational definition of ‘global health research’

Our research highlights the needs for a more consistent and operational definition of global health research. For example, we did not capture the full extent of research on the VSV-EBOV Ebola vaccine deployed in the emergency response to the March 2016 outbreak in our CRC and PubMed searches. This discrepancy may point to a divergence between the definitional scope of global health research and those researchers who either use this term, identify themselves as part of this epistemic community or actively participate within it. For example, whereas global health researchers may consider the developers of the Ebola vaccine to be global health researchers, Ebola vaccine researchers may not – instead considering themselves as infectious disease researchers, vaccinologists or virologists – and as a result may not be members of leading organisations of Canadian global health researchers (i.e. Canadian Coalition for Global Health Research) or attendees of the field’s conference (i.e. Canadian Conference on Global Health). One example of this discrepancy is the CIHR-funded CRC in Molecular Virology and Antiviral Therapeutics, Marceline Côté, whose CRC profile states that their chemical genomics and genetics research “*supports the development of antiviral therapies to fight emerging viruses, such as Ebola, and will help train the next generation of virology experts*” [26]. ‘Global health’, or ‘international health’, is not mentioned in their profile nor was their name captured in the CRC database search, but some may argue that clinical work on a transnational virus like Ebola would allow individuals to identify as ‘global health’ researchers. A standard and operational definition of global health research can help address the challenges we face in accurately mapping global health research expertise and support our quest for robust capacity-building.

#### Feasibly identifying a country’s global health research expertise

This method proved feasible and efficient for identifying a country’s research expertise. It relies on a logical framework of inputs, activities and outputs, and employs publicly available administrative datasets to identify the geographic and substantive foci of significant global health research expertise. In applying this method, it is clear that Canada has unique strengths and our findings have informed CIHR’s development of a strategic plan. In a tangible sense, these methods can be adapted and used to support research policy directives, including to inform national global health research strategies and to allow universities, civil society and research funding organisations to make more strategic decisions regarding their respective priorities and mandates.

### Strengths and limitations

This rapid environmental scan methodology allowed us to summarise existing data on Canada’s global health research expertise. Strengths of the method include its careful integration of a logical framework (one that considers a variety of inputs, activities and outputs), the use of publicly available data, and both methodological and data triangulation. The logical framework ensures that research inputs can be systematically attributed to research activities and research outputs that are relevant to each individual country. Using publicly available data reduces the barriers that arise from a lack of access. For example, other inputs include grants from industry yet, due to privacy laws, this type of information is not available publicly. The methodological triangulation and data triangulation ensure built-in redundancies that can help verify and challenge findings.

A limitation of this rapid environmental scan methodology is that it relies heavily on web searches and databases, which are only as accurate and up to date as the information posted on websites and databases. Institutions may be slow to update their websites; therefore, some of the information found may be outdated and could bias the findings to only the most recent items [27]. Web-based searches are also limited in their ability to identify webinars and other similar training sessions, typically biasing the findings to only the most recent training sessions. Databases may not follow a standard data collection protocol; for example, when applying this method to the Canadian context we found that some CRCs may not have been captured in our search because the CRCs did not have any information beyond their name or chair title uploaded for our search string to pick up. Additionally, relying on the Global Health MeSH in the PubMed database may skew the assessment of research productivity in favour of certain kinds of research outputs. The MeSH captures “*research on improving health and achieving equity in health for all people*” but would not capture all of the biomedical, clinical, health systems and epidemiological research relevant to the field of global health. Developing a better, optimised PubMed search string for global health research that was both highly sensitive and specific would have been better but was beyond the scope of this rapid scan.

## Conclusions

This study sought to develop a rapid environmental scan methodology to map where a country’s global health research expertise lies, both geographically and substantively. Our framework and three-pronged approach was highly effective in responding to the real-time evidence needs of decision-makers in Canada. Other researchers can build on this approach to map expertise and research strengths in other settings and, where timelines allow, may consider adding more indicators (e.g. global health conferences/workshops as activities or registered patents as outputs) or incorporating additional data sources, including surveys of universities and key-informant interviews with stakeholders.

## Supplementary information

**Additional file 1.** Workflow diagram of inputs, activities and outputs.

## Data Availability

All data generated or analysed during this study are included in this published article and its supplementary information files.

## Abbreviations

CIHR: Canadian Institutes of Health Research
CRC: Canada Research Chair
LMICs: Low- and middle-income countries
