# Supplementary figures and images for: Using a rapid environmental scan methodology to map country-level global health research expertise in Canada
Source: Health Res Policy Syst. 2020 Apr 9;18:37. doi: 10.1186/s12961-020-0543-x (PMC7146898; doi:10.1186/s12961-020-0543-x)

Additional file 1. Workflow diagram of inputs, activities, and outputs.


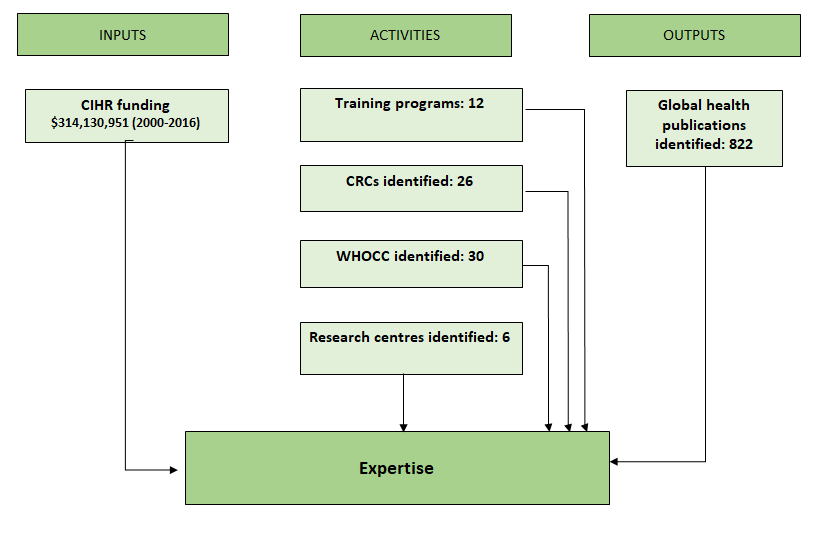

Supplement: Supplementary file 1 — Additional file 1. Workflow diagram of inputs, activities and outputs. [file 12961_2020_543_MOESM1_ESM.docx]
